# Deep-Learning-Based Segmentation of Keyhole in In-Situ X-ray Imaging of Laser Powder Bed Fusion

**DOI:** 10.3390/ma17020510

**Published:** 2024-01-21

**Authors:** William Dong, Jason Lian, Chengpo Yan, Yiran Zhong, Sumanth Karnati, Qilin Guo, Lianyi Chen, Dane Morgan

**Affiliations:** 1 Department of Mechanical Engineering, University of Wisconsin-Madison, Madison, WI 53706, USA; williamdong@berkeley.edu (W.D.); qilin.guo@unl.edu (Q.G.); 2 Department of Computer Science, University of Wisconsin-Madison, Madison, WI 53706, USA; jlian7@wisc.edu (J.L.); cyan46@wisc.edu (C.Y.); yzhong68@wisc.edu (Y.Z.); karnati2@wisc.edu (S.K.); 3 Department of Material Science & Engineering, University of Wisconsin-Madison, Madison, WI 53706, USA

**Keywords:** keyhole, laser powder bed fusion, deep learning, image segmentation

## Abstract

In laser powder bed fusion processes, keyholes are the gaseous cavities formed where laser interacts with metal, and their morphologies play an important role in defect formation and the final product quality. The in-situ X-ray imaging technique can monitor the keyhole dynamics from the side and capture keyhole shapes in the X-ray image stream. Keyhole shapes in X-ray images are then often labeled by humans for analysis, which increasingly involves attempting to correlate keyhole shapes with defects using machine learning. However, such labeling is tedious, time-consuming, error-prone, and cannot be scaled to large data sets. To use keyhole shapes more readily as the input to machine learning methods, an automatic tool to identify keyhole regions is desirable. In this paper, a deep-learning-based computer vision tool that can automatically segment keyhole shapes out of X-ray images is presented. The pipeline contains a filtering method and an implementation of the BASNet deep learning model to semantically segment the keyhole morphologies out of X-ray images. The presented tool shows promising average accuracy of 91.24% for keyhole area, and 92.81% for boundary shape, for a range of test dataset conditions in Al6061 (and one AliSi10Mg) alloys, with 300 training images/labels and 100 testing images for each trial. Prospective users may apply the presently trained tool or a retrained version following the approach used here to automatically label keyhole shapes in large image sets.

## 1. Introduction

Laser powder bed fusion (LPBF), also known as selective laser melting (SLM), is currently one of the most common metal additive manufacturing (AM) techniques [1,2]. During the LPBF process, a focused laser will shoot onto the powder bed selectively and make powders melt, merge, and solidify to build up a part based on the CAD (computer-aided design) model [3]. Using in-situ X-ray imaging to monitor the process, previous studies have found that high intensity of the laser will result in vaporization of the material, which will lead to recoil pressure that pushes the molten metal in the melt pool to create a vapor cavity, named a ‘keyhole’ [4,5]. In the process and along the printing path, the keyhole experiences inconsistent recoil pressure, surface tension, and Marangoni force, leading to severe and random fluctuations [6,7]. Sometimes, fluctuated keyholes will collapse, and the vapor will be partially trapped inside the melt pool, resulting in undesirable porosity in the final product [4,8].

Many studies have been made to find correlations between the keyhole morphology revealed in the X-ray imaging and the generation of keyhole-induced pores [9,10], with an eventual goal of reducing defects. Due to the complex dynamics of the LPBF process, there is great interest in in-situ data-driven methods to study the defect formation mechanisms [11,12]. As a popular approach to observe the dynamics in the LPBF process, in-situ X-ray imaging can be captured at a frame rate of over one million frames per second, generating enormous keyhole morphology images, and creating a huge potential for data-driven studies with keyholes. However, these studies are greatly constrained by a limited quantity of well-characterized keyhole morphology data, which typically needs to be labeled by humans. More specifically, sufficient data for machine learning-based pore prediction for many types of LPBF systems, conditions, and alloys, will require very large efforts to label keyhole morphologies unless accessible automatic tools that can segment the keyholes are developed. Several automatic tools have recently been explored in previous works: Pyeon et al. developed a non-machine learning-based semi-automatic keyhole region extraction tool [13], and Zhang et al. tested several semantic segmentation and object detection models and compared the performances of extracting keyholes and pores at the same time [14]. However, the filter developed by Pyeon et al. was only tested with clean images without metal powder, and models tested by Zhang et al. segment both keyholes and pores in the same classification. In addition, while the boundary is the most important feature in the keyhole morphology, previously proposed methods are not designed to have high segmentation boundary accuracy and be validated against datasets from different experiments. Therefore, considering the increasing need for keyhole segmentation for large X-ray imaging databases, we here develop an automatic keyhole segmentation tool with high accuracy for both area and boundary and test against datasets with powders from different experiments.

In this paper, a deep-learning-based semantic segmentation tool that is capable of automatic segmentation of keyholes in X-ray images with accurate boundaries was developed. This tool is composed of a filter that standardizes, normalizes, and cleans the X-ray images, and an implementation of BASNet, a Boundary-Aware Segmentation network, that predicts semantic labels [15]. Without any human inputs, this tool only requires users to run algorithms with their X-ray images, which significantly accelerates the keyhole morphology labeling process and enables the possibility of data-driven analysis with a large quantity of morphology data. In the following, the implementation of the method will be illustrated, the performance of the tool will be quantified, and the mechanism and limitation of the method will be discussed at the end. This work was conducted with data derived from different experiments at multiple different times, but the tool provided in this study will likely need to be refit for systems with significant differences from those studied here. However, such refitting can likely be performed quickly through transfer learning, i.e., by starting from the weights in this paper. Development of similar segmentation tasks for X-ray imaging, like segmenting melt pools or spatters in the LPBF process, could also be accelerated by transfer learning from the present model.

## 2. Methods

**Figure 1:**
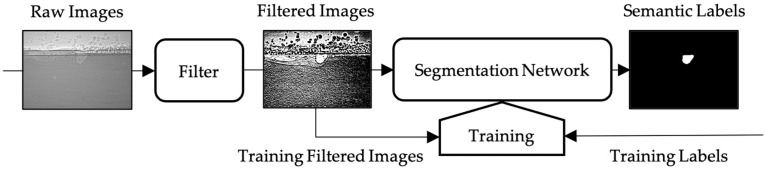
A flowchart illustrating the keyhole segmentation process pipeline.

The workflow of the whole segmentation process is shown in Figure 1: Raw X-ray images are processed with a designed filter and then fed into the segmentation network, which outputs the semantic labels. The segmentation network needs to be previously trained by filtered images and ground truth labels in the training set.

### 2.1. Raw Images

Data used in this study were acquired by using in situ X-ray imaging on LPBF processes at the beamline 32-ID-B of the Advanced Photon Source at Argonne National Laboratory. During experiments, the ytterbium fiber laser generated a laser beam and was directed by a galvo scan head toward the metal powder and substrate. While the laser was melting the material, the scanning area was penetrated by X-ray simultaneously, and the shapes of keyholes and pores were projected and converted to visible light by a scintillator, which was recorded by a high-speed camera with a frame rate of 50 kHz [4]. There are 8 X-ray imaging data sets in this work and they were acquired by 8 separate experiments, each with different processing parameters, as shown in Table 1. The experiments were performed with Al6061 in 7 cases and AliSi10Mg in one case. Each data set has 400–500 frames, and 50 of those were labeled in each data set and used in this study. In total, there are 3416 images in the 8 data sets from which 1441 images have a visible keyhole, and 400 images with visible keyholes were labeled for training and testing. The remaining 1975 images show no keyholes since the laser in the LPBF process was not on or in the field of view at the time of imaging, and are not used in the testing and training of this paper.

### 2.2. Filtering

**Figure 2:**
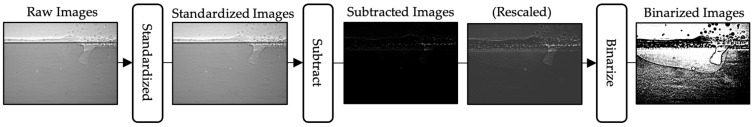
Flowchart illustrating filtering procedures. The subtracted image is rescaled for better representation in the following image, no re-scaling was involved in the “Subtract” and “Binarize” steps.

There are two major goals when filtering the raw images: (1) standardize images to be acceptable for the segmentation network as inputs, and (2) normalize and clean the image by reducing the differences between datasets and removing stationary obstructions. The filter is built using MATLAB R2023b based on the schematics shown below in Figure 2 and described below.

To meet the requirements of the network, all images need to be at the same resolution, a fixed size, and in the same format. In our case, all images are converted into uint 8, with a size of 700×500 pixels, and in PNG format. This step is known as “Standardize”.

In different experiment setups, X-ray images will have different brightness, contrast, and sometimes stationary obstructions overlapping with the keyhole area. These factors greatly hinder the segmentation of keyholes. To alleviate these factors, a concurrent subtraction step is designed, and the detailed mechanism will be covered in the Discussion section. For each image, the sum of previous N frames of images is calculated, and the current image is subtracted by the average of previous N frames, where N is a tunable parameter for each dataset, and here 40 previous images are used. This step is termed “Subtract”.

To further normalize the images, greyscale images are converted into black and white (binary) images. The images are binarized with a threshold of 0.5. The global average value of all pixels in the whole image is calculated, pixels with a value above the average are marked as 1, and pixels with a value below average are marked as 0. This step is “Binarize”.

With these three steps, any raw X-ray images can be transformed into the format used for training and can be input into our training model. Please check the GitHub repository in the Supplementary Material to find a specific implementation of these steps.

### 2.3. Segmentation Network Training

Before the filtered images are fed into the segmentation network, the network needs to be trained by filtered training images and corresponding ground truth labeling. The ground truths are labeled manually using MATLAB R2023b Image Labeler app, with pixels assigned as 1 in the keyhole region and pixels assigned as 0 in the background, exported with the same resolution, size, and format as training images, creating a binary label for each corresponding image. In this study, experiments/datasets 3 to 8 were picked as training datasets, and their corresponding ground truth labels were picked as the training set, with 50 images and labels for each of the 6 datasets, and 300 images and labels in total.

In this study, we chose the Boundary-Aware Segmentation Network (BASNet) developed by Qin et al. as the semantic segmentation network [15]. The BASNet model was trained at a batch size equal to 1, and 70 epochs, leading to 21,000 iterations in total, and the trained model can be found in the GitHub repository in the Supplementary Material. The model appeared to be well-converged by this number of epochs and the loss curve can be found in the Results and Discussion section. The source code for training was modified to add a testing step with datasets 1 and 2 after each epoch, where the model was tested to generate the labels for images in datasets 1 and 2. The labels were compared with the ground truths and the testing losses for these two datasets were also calculated after each epoch of training. Convergence on testing loss was also observed, with more details in the Results and Discussion section.

### 2.4. Deep Learning Segmentation on Test Data

**Figure 3:**
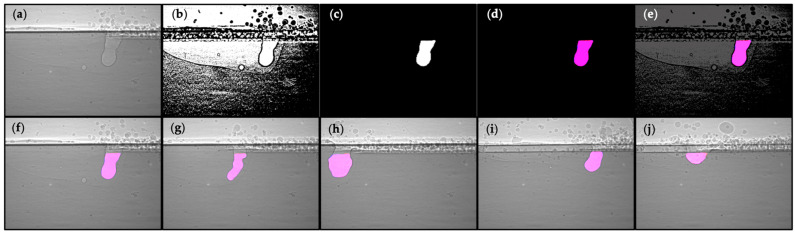
Sample X-ray image, its predicted label, and comparisons: (**a**) raw image; (**b**) filtered image; (**c**) predicted label; (**d**) overlay of predicted label and ground truth, where predicted label is in red, ground truth is in blue, intersection is overlapped as purple (Since there are not much unmatched predicted label and ground truth, red and blue area are barely visible for the given images); (**e**) overlay on filtered image; (**f**) overlay on raw image; (**g**,**h**) overlay on raw images for other two frames in testing dataset 1; (**i**,**j**) overlay on raw images for two frames in testing dataset 2.

Experiments/datasets 1 and 2 were picked as the testing datasets for the segmentation model, and the ground truth was labeled in advance as a comparison to prediction. Filtered X-ray images for datasets 1 and 2 were fed into the trained BASNet model to generate predicted labels, which need to be binarized to convert the label from gradient to binary images. The sample X-ray image, its predicted label, and comparisons with ground truth are shown in Figure 3.

Testing on data from different experiments can allow for evaluation of the actual performance of this tool when prospective users implement this tool to acquire keyhole morphology on their own X-ray images. The performance of this model on testing datasets will be quantified in the Results section.

## 3. Results

### 3.1. Training and Loss Function

**Figure 4:**
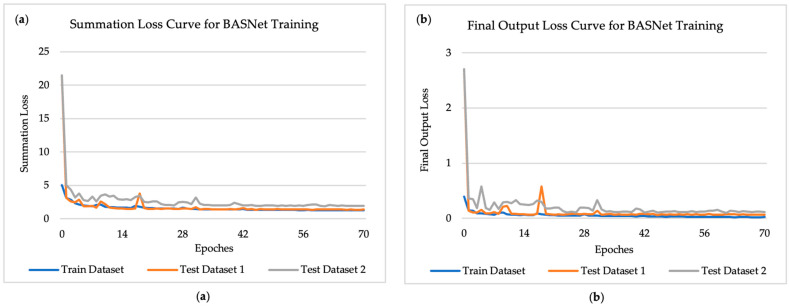
Recorded loss curve for BASNet training process for training datasets (experiment 3–8), test dataset 1 and 2, from 0 epoch to 70 epochs: (**a**) training and testing data loss curve for summation loss; (**b**) training and testing data loss curve for final output loss.

The BASNet model adopts a predict-refinement structure, where the input image first passes through a predict module (encoder–decoder) and then a residual refinement module to finally generate the segmentation. The segmentation generated by the refinement module is the final output of the model, and there are 7 “side outputs”, or intermediate outputs, which are the outputs of every stage of the decoder in the predict module, and are also the inputs of their upcoming stage. The loss function for optimization of BASNet takes the summation of loss values of all final outputs and 7 side outputs generated along the network, which is named “summation loss”. While this is useful for training, the performance a user cares about is the loss from the final output, which is named “final output loss”, as that is what will be used in applications. Both training summation loss and the final output loss for the training and test data were recorded at the end of each epoch, shown in Figure 4 below. As the training loss curve shows in Figure 4a, the model gradually converged to a low and consistent summation loss value as training proceeded. The model also performed nearly as well on test datasets, which also gradually converged to a low loss value along with the training loss. During training, the final output loss for the training datasets and test datasets was also calculated and is shown in Figure 4b below. The convergence of the training and test datasets onto low loss values indicates the high accuracy of segmentation of the trained model on both training and test datasets.

### 3.2. Testing and Performance Matrices

To evaluate the performance of the tool, by comparing with the predicted label and ground truth, two matrices are calculated to quantify the segmentation accuracy of the pipeline: intersection over union (IoU) and boundary F-score (BF-score) (both defined below). Both IoU and BF-score are in the range [0, 1], an IoU closer to 1 means a better match in area, and a BF-score close to 1 means a better match on the boundary [16]. These two metrics are calculated based on the predicted label and ground truth for all images in both testing datasets 1 and 2, based on Equations (1) to (4) below, where PL and PLB stand for the model-predicted label and its boundary, and GT and GTB stand for the ground truth label and its boundary, which represents the actual keyhole region. In the IoU calculation, the intersection area and union area of the predicted label and ground truth are calculated, and a ratio of intersection over union that is close to 1 shows both successful coverage of the actual keyhole area by the model predicted labeling and little overestimation of the actual keyhole area by the predicted labeling. In the BF-score calculation, precision represents the percentage of the model-predicted boundary that matches the actual keyhole boundary, and high precision means the model predicted a more correct boundary. Recall represents the percentage of the actual keyhole boundary that is predicted correctly by the model-predicted boundary, and high recall indicates more actual keyhole boundary is successfully predicted by the model. The BF-score is calculated by the multiplication over the sum of the precision and recall times by 2, and a score close to 1 indicates both high precision and recall by the model prediction. In Equations (2) and (3), the threshold is set for the maximum distance between two boundaries is 1 pixel, meaning that any portion of the boundary that exceeds 1 pixel distance from the other will not count in the numerator. This is a demanding criterion, representing just a fraction of a percent of the dimensions of the filtered images, which in this study are 700 by 300 pixels.

IoU = (Area Intersection of PL and GT)/(Area Union of PL and GT)(1)

Precision = (Portion of PLB with distance to GTB within the threshold)/(Full PLB)(2)

Recall = (Portion of GTB with distance to PLB within the threshold)/(Full GTB)(3)

BF-score = 2 × (Precision × Recall)/(Precision + Recall)(4)

As shown in Table 2 below, IoU and BF-score are high and close to 1 for both datasets 1 and 2 for run 1, which means that this method successfully segments out the keyhole region in test datasets. Considering IoU, this method is on the same level as other segmentation tools proposed in previous works, and a high BF-score further validates the segmentation accuracy on the boundary [14]. The same tool is later tested using cross-validation, with three more runs trained and tested as Table 3 below. The IoU and BF-score for cross-validation are also shown in Table 2, with most testing datasets showing similar values for both matrices, with an average IoU of 0.9124 and average BF-score of 0.9281, suggesting that this tool is very accurate for random 75% training/25% test splits. However, the proposed tool might also encounter segmentation errors, as testing dataset 3 for run 2 shows relatively lower IoU and BF-score values, which will be further covered in the Discussion section.

## 4. Discussion

We constructed a pipeline for keyhole region semantic segmentation in in-situ LPBF X-ray Imaging. The whole pipeline consists of two main components, a filter that standardizes, normalizes, and cleans raw datasets, and deep learning segmentation labels the keyhole region from the filtered images. In different experiment setups, X-ray images have different brightness, contrast, and sometimes stationary obstructions overlapping with the keyhole area. These factors greatly hinder the segmentation of keyholes.

Therefore, the intuition of the design of the filter part in the pipeline is to manage all training data in a consistent fashion, so as to reduce the effects of attributes of the datasets on the prediction of keyhole segmentation. From the eight raw datasets used in this experiment, images from four datasets have a dimension of 712×512 pixels, and images from four other datasets have a dimension of 896×448 pixels, all in TIF format. Theoretically, having images with different sizes should still be applicable for training, as there is a rescaling step in the training algorithm, but a consistent size and file format will make image labeling and manipulation much easier. Hence, the designed Standardize step converts all images into 700×500 pixels and PNG format.

As shown in Table 4, raw images from different datasets have vastly different brightness and contrast values, and the differences can be mitigated with the Subtract and the Binarize step to normalize images from all datasets. Firstly, the Subtract step greatly reduces the differences by subtracting the average image of the whole dataset, leaving only the features of each image relative to the dataset. Then, the differences are further alleviated by the Binarize step, which reduces the greyscale difference across different datasets by turning the image into black and white, which will have contrast of 1.

**Figure 5:**
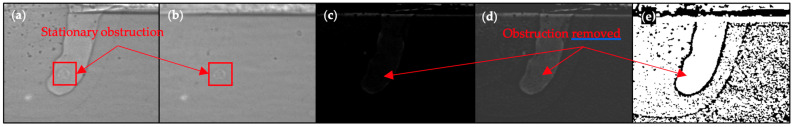
The Subtract step in the filter removes a stationary obstruction affiliated to the dataset: (**a**) a standardized image with the obstruction inside the keyhole; (**b**) the subtraction value as an image, average of 40 previous images in the same dataset, containing the obstruction; (**c**) the subtracted image, where the obstruction is removed by the Subtract step; (**d**) the rescaled subtracted image for visualization; (**e**) binarized image, the output of the filter, with no obstruction.

Despite brightness and contrast, stationary obstruction also greatly hinders the segmentation of keyholes, especially when they are spatially overlapped with the keyhole area. These obstructions are stationary and are affiliated with a particular dataset, which yields inconsistent X-ray images across different datasets and influences the prediction of the keyhole area. As shown in Figure 5, the obstruction can also be resolved with the Subtraction step, as the subtraction value shown in Figure 5b contains the stationary patterns, and stationary obstructions can be removed for subtracted and binarized images, as shown in Figure 5c–e. Note that the deep learning model segmentations described below with just the Standardize step and just the Subtract step were also tried, but the model did not perform well and failed to identify the keyhole region in most frames.

For the deep learning segmentation, BASNet utilizes a hybrid fusion loss function to achieve training supervision of prediction on multiple levels: pixel level, patch level, and map level, instead of barely relying on IoU, which could lead to insufficient prediction of structural properties on the patch level. Along with the prediction-refinement structure, BASNet is capable of semantic segmentation for boundary-sensitive cases like the detailed morphology in keyhole segmentation. The boundary prediction performance is further validated with the BF-score in the Results section. Other popular models like UNet and Deep LabV3+ were also tried in the pipeline, but they all failed to map the boundary in most cases even when with a high IoU score [17,18].

**Figure 6:**
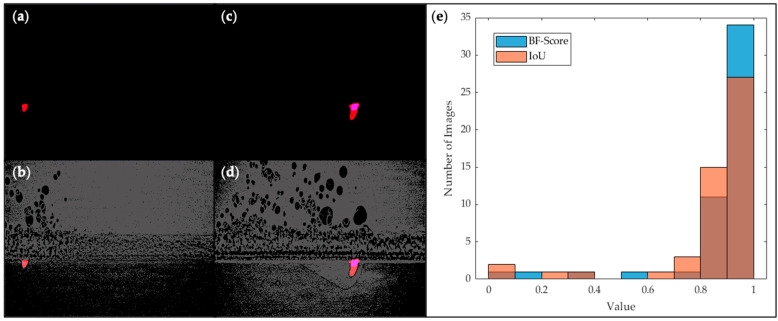
Segmentation errors in run 2, testing dataset 3: (**a**) overlay of the predicted label and ground truth of a failed segmentation example, no label is predicted by BASNet (predicted label in blue, ground truth in red, intersection in purple); (**b**) overlay on the input filtered image; (**c**) another failed segmentation example, where the “tail” of the keyhole region is missed by prediction; (**d**) overlay on the input filtered image; (**e**) histogram of the distribution of tested images in testing dataset 3 in run 2 regarding two evaluation matrices (Number of images with respect to BF-Score in blue, IoU in red, and overlapped in darker orange). Blue area in (a–d) is barely visible because there is little predicted area that is not ground truth.

However, the proposed tool may also encounter segmentation errors, like run 2 testing dataset 3 mentioned in the Results section. Two failed segmentation examples are shown in Figure 6, where in the first example (Figure 6a,b), BASNet predicts no keyhole region, resulting in an IoU and BF-score of less than 0.1. In the second example, shown in Figure 6c,d, BASNet failed to predict the “tail” of the keyhole region. As shown in Figure 6e, a small number of similar errors contribute to the relatively low average IoU and BF-score, while in most cases, BASNet can accurately label the keyhole region. These errors could be attributed to the small keyhole size of dataset 3, which leads to inconsistency in the prediction area with other datasets. Consistency can potentially be achieved by adding a cropping module in the filter or introducing more training data with similar sizes to reduce the effect of keyhole size on the prediction of BASNet. In addition, another factor behind segmentation errors is the fuzzy imaging background for dataset 3, which results in unclear keyhole region contour of the filtered images. Further fine-tuning of the filter parameter N could potentially improve the clarity of the image with a distinct keyhole boundary.

So far, the current algorithm cannot process X-ray images with significant differences in experiment setups, and re-training might be needed for prospective users to implement the algorithm. In the future, more training images will be labeled to further optimize this algorithm and to enhance the versatility and robustness of the model.

## 5. Conclusions

In this study, a deep-learning-based segmentation tool that is capable of automatic segmentation of keyhole morphology in X-ray images with a filter and a trained network pipeline was developed. This tool is validated, with an average IoU of 0.9124 and an average BF-score of 0.9281 on X-ray images from different experiments, proving its high accuracy both in area and boundary, with cross-validation of 300 training and 100 testing images/labeling for each trail.

This work illustrates a repeatable approach for prospective users to automatically generate massive keyhole morphology data with high accuracy on area and boundary from X-ray images. Sufficient morphology data will support developing data-driven analysis of LPBF processes to further improve the quality of additively manufactured products.

## Figures and Tables

**Table 1:** Samples and processing parameters of in situ X-ray imaging experiments.

Experiment	Material	Nanoparticle	Substrate	Power (W)	Scan Speed (m/s)
1	Al6061	10%vol TiC	Printed	385	0.3
2	Al6061	10%vol TiC	Printed	385	0.4
3	Al6061	/	Printed	443	0.4
4	Al6061	/	As-cast	500	0.2
5	Al6061	/	Printed	500	0.4
6	Al6061	/	As-cast	500	0.4
7	Al6061	/	As-cast	500	0.4
8	AlSi10Mg	/	As-cast	500	0.5

**Table 2:** IoU and BF-score for cross validation with 6 training sets and 2 testing sets, 4 runs in total.

Run	Testing Dataset	IoU	BF-Score
1	1	0.9381	0.9514
1	2	0.8923	0.9098
2	3	0.8333	0.8603
2	4	0.9321	0.9421
3	5	0.9195	0.9352
3	6	0.9190	0.9351
4	7	0.9130	0.9313
4	8	0.9518	0.9595
**Average**		**0.9124**	**0.9281**

**Table 3:** Cross validation training and testing datasets assignments.

Run	Dataset 1 and 2	Dataset 3 and 4	Dataset 5 and 6	Dataset 7 and 8
1	Test	Train	Train	Train
2	Train	Test	Train	Train
3	Train	Train	Test	Train
4	Train	Train	Train	Test

**Table 4:** Average brightness and average contrast value for raw, subtracted, and binarized images for all images in 8 datasets. For each image, brightness is calculated by the mean of all pixels’ value over the white value (255 for uint 8), and contrast is calculated by the range of pixels’ value (maximum − minimum) over the white value. Values from all 50 images for each dataset are averaged.

Dataset	Average Brightness			Average Contrast		
	Raw	Subtracted	Binarized	Raw	Subtracted	Binarized
1	0.5852	0.0079	0.0363	0.8034	0.4820	1.0000
2	0.5802	0.0091	0.0374	0.7496	0.4958	1.0000
3	0.0131	0.0001	0.0314	0.0278	0.0155	1.0000
4	0.0113	0.0001	0.0275	0.0255	0.0129	1.0000
5	0.0131	0.0001	0.0328	0.0288	0.0156	1.0000
6	0.5841	0.0097	0.0350	0.7833	0.5260	1.0000
7	0.0115	0.0001	0.0215	0.0264	0.0119	1.0000
8	0.5876	0.0083	0.0389	0.7503	0.4261	1.0000

## Data Availability

The computer source code is publicly available at: https://github.com/WilliamDongSH/KeyholeSeg. Data used in this study is available from the corresponding author upon reasonable request.

## Supplementary Materials

The following supporting information can be downloaded at: https://github.com/WilliamDongSH/KeyholeSeg, Computer Source Code S1: MATLab filter for keyhole segmentation; Computer Source Code S2: MATLab checker for keyhole segmentation accuracy performance; Computer Source Code S3: Modified BASNet code with loss visualization and logging; Computer Source Code S4: Trained BASNet model.
